# Book Reviews

**Published:** 1888-01

**Authors:** 


### BOOK-REVIEWS.

On the Animal Alkaloids. The Ptomäines, Leucomäines and Extractives in Their Pathological Relations. By Sir William Aitken, Knt., M. D., F. R. S. Pp. vi and 61. 1887. Philadelphia: P. Blakiston, Son & Co. Chicago: W. T. Keener.

The aim of this litte work is, perhaps, best shown by a portion of the author's preface. He says: "The following pages owe their existence to the necessity of finding a subject suitable for a lecture introductory to the course of instruction at the Army Medical School at Netley." "Speaking generally, it may be said that the investigations which underlie the most important practical work of the military medical officer, alike on land and sea, relate especially to the causation of diseases, and with a view of their prevention; and in carrying out such investigations, the time appears to me to have come when it is desirable to open up new lines of thought and of practical departure in pathology, which may lead us to entertain broader, or at any rate less narrow, views than those we have been accustomed to entertain as to the origin of some diseases." * * * "So much has been already done by experiment and research in the past twelve or fifteen years, that such a concise summary as I have here attempted to give may show the bearing of the results and their value, as illustrating the pathology and the origin of some diseases.

"It is further desirable, if possible, to get rid of the term 'cause' altogether, as applicable to any particular disease. Our text-books, as yet, have been unable to specify and establish any *single* thing as the final cause of any disease. There is no disease I know of which acknowledges a *single* cause. It ought rather to be our business to find out the many and ever varying factors or conditions which, as antecedents, combine to produce disease; and while we must acknowledge the influence of many physiological agents in aiding and abetting these factors, we must mainly look to the physiological agencies within our own bodies during life as competent to bring about many forms of disease."

The author first considers the *Elaboration of Alkaloids from proteid substances* (Ptomäines and Leucomäines); and also of *azotized uncrystallizable substances* (extractives) and their toxic properties.

He then considers the *Pathology and Symptoms of Poisoning* by the extractives and by the animal alkaloids—poisoning by the accumulation of normally elaborated but toxic products, and the origin in this way of constitutional diseases.

Much of the address is based upon the results of the investigations, as the author states, by MM. Gautier and Peter, and Drs. A. M. Brown and Lander Brunton. He says: "May we not, therefore, recognize in these affections the evidence of poisoning by a toxic alkaloid, or alkaloid of putrefaction; and that certain nutritive ingesta may give rise to the formation of such toxic alkaloids?" and then quotes from Dr. A. M. Brown: "Hence it is no great stretch of scientific license for us to recognize or trace in pathology a very natural series of morbid phenomena or ailments starting from the *botulism* of the Germans up to Asiatic cholera."

The address contains useful and practical suggestions, and its careful perusal will be found to be interesting.

Syphilis. By Jonathan Hutchinson, F. R. S., LL. D. With eight chromo-lithographs. Pp. xii and 532. Philadelphia: Lea Brothers & Co. Chicago: A. C. McClurg & Co.

This small volume is one of the *Clinical Manuals for Practioners and Students of Medicine*, issued by Messrs. Lea Brothers & Co.

The author of this manual is so well known, and justly held in so high esteem by the medical profession, both of Europe and America, that whatever emanates from his pen commands attention, for it is always safe to assume that it will not be found to be wanting in merit. He has given so much attention to the subject of syphilis as to have made him a recognized authority on that disease. His name is probably permanently connected with that deformity of the teeth known as *Hutchinson's teeth*, one of the marked evidences of inherited syphilis, which he so accurately described as an almost pathognomonic symptom of that affection. In his preface to this manual he says: "The literature of syphilis is encumbered with ill-founded opinions and untrustworthy facts." "Our patients often have reasons for not telling us the exact truth, and, still more often, they are not themselves cognizant of it." "None of the symptoms of the disease are pathognomic, and with the best desire in the world to be candid, both patients and their advisers may give us misleading evidence. Such being the sources of error, it becomes wise to distrust all isolated facts, however definite they may appear to be," etc., etc. Such words, from such an authority, should have great weight with the large number of physicians who are called upon to treat this disease in isolated cases, where the statements of patients cannot be implicitly accepted for the reasons given in this preface, and should emphasize the necessity for the greatest care in diganosis, treatment and prognosis.

The colored plates are intended to represent:

I. Syphilitic Choroiditis.
II. Erratic Chancres on the Fingers and on the Eyelid.
III. Vaccination Chancres.
IV. Chancre of the Tongue, and Papillary Growths on Tongue.
V. Chancre of Nipple, and Syphilitic Disease of Tongue.
VI. Teeth of Inherited Syphilis.
VII. Different Stages of Syphilitic Keratitis (Inherited).
VIII. Syphilitic Disease of the Nails.

These chromo-lithographs are fairly well executed, and are not of the kind sometimes seen in medical works, which are effective in misleading.

The character of the work and the price merited better binding than that in which it is issued, and which can scarcely be said to be in keeping with the manner in which this long-established house is accustomed to issue books.

FUNCTIONAL NERVOUS DISEASES—THEIR CAUSES AND THEIR TREATMENT, WITH A SUPPLEMENT ON THE ANOMALIES OF REFRACTION AND ACCOMMODATION OF THE EYE, AND OF THE OCULAR MUSCLES. By GEORGE T. STEVENS, M.D., Ph. D. Pp. xiii and 217. New York: D. Appleton & Company. Chicago: A. C. McClurg & Company.

The author says: "This memoir * * * is now presented in the English, as it was then in the French language," when it was awarded a prize by the Royal Academy of Medicine of Belgium. Some changes and some additions, which are noted, have been made in the present edition.

It deals with a practical subject, which may be said to be a sort of connecting link between neurology and ophthalmology, since many of the reflex nervous troubles are connected, either as cause or consequence, with abnormalities of the eye or its appendages. Recognition of this fact, in the last few years, has led to the relief of much suffering from functional disorders originating in errors of refraction and defects of accommodation. Whether the positive and well-known views of the author of this book regarding the influence of the action of the muscles of the eyeball be accepted in their entirety or not, it is due him to say that he has been an industrious investigator of the subject, and that his work has contributed to a better understanding of it. His book is a fair exposition of his views, and it is no small compliment to him that so eminent a body of medical men as the Royal Academy of Medicine of Belgium should have awarded the work such recognition.

Asthenopia, like neurasthenia, is often a result, following from one or more causes, so that careful investigation is generally necessary to discovery of the origin of it. With the tendency of the human mind toward what are regarded as *hobbies*, it becomes the physician to be on guard against acceptance of the extreme views sometimes held by enthusiasts; but it is well in our efforts to avoid the Scylla of enthusiasm that we should not fall into the Charybdis of indifference. Even if this work does make claims which some may be slow to accept, its suggestiveness will serve a good purpose, and all will be glad to accord him whatever credit may properly attach to his claim of priority in recognition of the causes and their influence in these cases of asthenopia and reflex troubles if longer time should lead to full acceptance of his views by the medical profession. Whatever view may be taken of the advanced views of the author, the book will probably serve a good purpose.

HEALTH LESSONS. A PRIMARY BOOK. By JEROME WALKER, M. D. Pp. 194. 1887. New York: D. Appleton & Company. Chicago: A. C. McClurg & Company.

"In this little book, the aim has been to teach health subjects to young children in a truthful and interesting way, and by somewhat different methods than those usually employed."

The aim is a commendable one, and it is sought to accomplish that object at an important time in life. One can but wish that the effort might be successful. If children could be made to understand more about their bodies and the requirements of them, and the influence exerted by environment, the resulting tendency would be in the direction of better mental and physical health, and life would be entered upon under more favorable circumstances. With better health, there would be less temptation in the later years of life to the use and abuse of nervous stimulants. A glance at the titles of the seventeen chapters of which the book treats, in a unique manner, will give a fair idea of the field the author seeks to cover. The mode of presentation of the subjects, and the illustrations given, are likely to attract the attention of children, and to interest them.

I. Our Bodies.
II. What our Bodies Need.
III. Air and Sunlight.
IV. Breathing, Voice, Air-Supply.
V. Use and Abuse of Food.
VI. How Food Becomes Blood.
VII. What to Eat.
VIII. Wear and Repair.
IX. Warmth and Clothing.
X. Cleanliness.
XI. Work and Play.
XII. Our Framework.
XIII. How we Move.
XIV. Rest
XV. The Brain and the Nerves.
XVI. The Senses.
XVII. Accidents, Injuries and Poisons.

MANUAL OF CLINICAL DIAGNOSIS. By OTTO SEIFERT AND Dr. FRIEDRICH MÜLLER. Third Edition. Revised and Corrected by Dr. FRIEDRICH MÜLLER. Translated by WILLIAM BUCKINGHAM CANFIELD, A. M., M. D. With sixty illustrations. Pp. xi and 173. New York and London: G. P. Putnam's Sons. Chicago: W. T. Keener.

In the preface to the first edition (1886) of this manual, the authors say: "We have endeavored to supply a want by giving, in an epitomized form, the different methods of examination." "We have also endeavored to consider the practical needs of the student and physician by noting only what is reliable, and omitting everything self-evident and of secondary importance."

In the preface to the second edition (1886) it is stated that "a number of improvements and additions have been made, and among them it seemed necessary to add some new illustrations, especially to the chapters on blood and urine."

In his preface to the English translation of the book (1887) it is stated that "it has been brought down to the latest acquisitions of science, thus representing the most advanced views."

The work is divided into fourteen chapters on *The Blood*, *The Temperature*, *Organs of Respiration*, *The Sputum*, *Circulatory System*, *The Pulse*, *Digestive and Abdominal Organs*, *The Urine-Producing System*, *Transudations and Exudations*, *Parasites* (animal and vegetable), *The Nervous System*, *Analysis of The Pathological Concrements*, *Metabolism and Nutrition.* There are also added a *Table of the Weights of The Human Body*, and a Dose Table.

The illustrations, with the exception of some of the tracings of the pulse, are good. It is well printed, on good paper, and is a useful little book.

A MANUAL OF THE PHYSICAL DIAGNOSIS OF THORACIC DISEASES. By E. DARWIN HUDSON, JR., A.M., M.D., late Professor of General Medicine and Diseases of the Chest in the New York Polyclinic; Physician to Bellevue Hospital, etc. One volume. Octavo. 162 pages. Nearly 100 illustrations. New York: William Wood & Company. Chicago: W. T. Keener.

This handbook, by the late Dr. Hudson, designed originally for the use of his followers in the New York Polyclinic, is presented in its present form, enlarged and amended to meet the requirements of practitioners and advanced students.

The regional anatomy of the chest, the methods and instruments of precision used in its exploration, the acoustics of normal and abnormal chest-sounds, and the normal and pathological condition upon which these depend, are briefly, but comprehensively, reviewed. The intra-thoracic diseases of the respiratory tract are presented very conveniently for reference in tabulated synopses, embracing under each the definition, causes, symptoms, physical signs, diagnosis, prognosis and treatment.

The normal and abnormal states of the heart are similarly treated, and a short tabulated list of cardiac organic and inorganic murmurs is added.

In so small a work, upon such a comprehensive subject, it is impossible to make more than a brief allusion to many important items, and much that is of interest must necessarily be omitted. This little volume will be found useful by many as a convenient manual of the clinical aspects of intra-thoracic diseases.

A MANUAL OF ORGANIC MATERIA MEDICA. Being a Guide to Materia Medica of the Vegetable and Animal Kingdoms, for the use of Students, Druggists, Pharmacists and Physicians. By JOHN M. MAISCH, Phar. D., Professor of Materia Medica and Botany in the Philadelphia College of Pharmacy. Third Edition. With two hundred and fifty-seven illustrations. Pp. xv, 531. Philadelphia: Lea Brothers & Co. Chicago: A. C. McClurg & Co.

The intention has been to produce a text-book embracing in a concise form, the essential physical, histological and chemical characters of organic drugs, for use in connection with lectures in colleges of pharmacy. That the work has rapidly reached its third edition is evidence of appreciation, and none will question the judgment of the author, who is well known in connection with the National Dispensatory, concerning the requirements of these students, and of pharmacists generally. An epitome often suffices to recall the details of the lecture. The illustrations are numerous and excellent.

It is, however, not so well adapted to the needs of students of medicine, for the reason that the medical properties of drugs are but superficially and sometimes inaccurately described, and officinal preparations with their doses are omitted.

W. E. C.

DRUITT'S SURGEON'S VADE-MECUM. A MANUAL OF MODERN SURGERY. Twelfth Edition. 1887. Octavo. Pp. 985. With three hundred and seventy-three illustrations. Philadelphia: Lea Brothers & Co. Chicago: A. C. McClurg & Co.

In its new edition this work is much enlarged, and radical changes have been made in arrangement of the text, as well as in the treatment of the subject-matter.

The editor has incorporated most of the newer ideas of pathology. The pages on diagnosis constitute a new and excellent feature.

Many of the illustrations are new and graphic, being drawn from life.

In the opening chapters bacteria are classified, and their causative relation to disease discussed.

The pathology and classification of infective diseases are those of the latest investigators.

In short, the book is modern, clearly written, well arranged, and much more complete than most manuals.

It will be a valuable addition to the students' list of text-books. H. H. F.

THE VEST-POCKET ANATOMIST. (Founded upon "Gray.") By C. HENRI LEONARD, A. M., M. D. Thirteenth Revised Edition. Eighty-five illustrations. Pp. 154. Detroit: The Illustrated Medical Journal Company.

The fact that this small handbook of anatomy has reached a thirteenth edition would indicate that there has been a demand for even so condensed a work on anatomy.

The illustrations are fairly good copies of those given in Gray's Anatomy, and the size of the book makes it convenient for the student.

THE URINE. MEMORANDA, CHEMICAL AND MICROSCOPICAL, FOR LABORATORY USE. By J. W. HOLLAND, M. D. Illustrated. Pp. vi and 43. Philadelphia: P. Blakiston, Son & Co. Chicago: W. T. Keener.

This small manual, designed "For Laboratory Use," will be found to be of convenient size for that purpose.

It first gives the *composition of healthy urine*, which is followed by examination of morbid urine. After instruction as to the method of collecting the urine to be examined, especially that secreted during twenty-four hours, when accurate results are desired, directions are given for the various tests. These are accompanied by cuts, which are quite accurate, showing the different kinds of apparatus in making these tests, and seventeen illustrations of characteristics of morbid urines.

The paper and press-work are good, and the binding is as good as could be expected in a book that costs but fifty cents.

